# 

**Published:** 1898-07

**Authors:** 


### CONTENTS.

PAGE

ORIGINAL ARTICLES:
The Progress of Veterinary Science. By GEORGE HILTON, V.S. . . . . . 441
Puerperal Septicæmia (Parturient Apoplexy, Milk-fever, etc.). By J. L. CLARK, V.S. 449
Roup or Canker: Cause and Treatment. By H. A. STEVENSON, M.D., C.M., . 451

ABSTRACTS FROM FOREIGN JOURNALS:
Determination of Incipient Tuberculosis with the Aid of Roentgen Rays . . . . 457
Eucaine: A New Local Anæsthetic . . . . . . . . . . . 458
Ophthalmoscopic Course for Army Veterinarians . . . . . . . . 458

SELECTIONS:
Aphthæ, or Vesicular Stomatitis of the Horse. By D. HUTCHEON . . . . . 459
Lard as an Antidote for Strychnine-poisoning. By W. D. TURNER, M.D. . . 461

THERAPEUTIC NOTES:
The Comparative Value of Antiseptics . . . . . . . . . . . 463
Internal Administration of Creosote . . . . . . . . . . . 463
To Destroy Worms in Powdered Vegetable Drugs . . . . . . . . 464

REPORTS OF CASES:
Multiple Sclerosis in a Dog. By WILFRED LELLMAN, D.V.M. . . . . . 466
Twist, or Rotation of the Colon. By THOMAS V. SIMPSON, V.S. . . . . . 468
Osteomyelitis Generalis. By A. B. CAMPBELL, V.S. . . . . . . . . 469
Impaction of Rectum. By H. E. JAMES, V.S. . . . . . . . . . 471
Abnormal Position of the Kidney. By S. J. J. HARGER, V.M.D. . . . . . 471
Hæmaturia from Umbilical Vein; Constipation. By O. W. GRAHAM, V.S. . . 472
An Unexpected Recovery. By JAMES and PERLEY, V.S. . . . . . . . 473
Clinic Notes from the McKillip Veterinary College . . . . . . . 474

AMONG THE PROFESSION IN CANADA . . . . . . . . . . 475
Canada Veterinary Associations—Veterinary Department of McGill University—Among Montreal Graduates—Massachusetts Alumni Association of McGill Veterinary Department—Ontario Veterinary College—Among the Graduates of the Ontario Veterinary College—Livestock Inspectors—Notes and Personals 475–487

EDITORIALS:
Our Canada Edition . . . . . . . . . . . . . . . 488
An Excellent Appointment . . . . . . . . . . . . . . 488
Manitoba, the Realm of Veterinarians . . . . . . . . . . 489
Seventh International Congress of Veterinary Surgeons at Baden-Baden, 1899 . . 491
Journal Special to the U.S.V.M.A.—Omaha, September 6-9, 1898 . . . . . 492

ARMY DRIFT . . . . . . . . . . . . . . . . . . 494

SOCIETY PROCEEDINGS . . . . . . . . . . . . . . . 498

COMMENCEMENT EXERCISES . . . . . . . . . . . . . 501

PERSONALS—POINTS . . . . . . . . . . . . . . . 510–514

